# A Modified Murine Calvarial Osteolysis Model Exposed to Ti Particles in Aseptic Loosening

**DOI:** 10.1155/2020/3403489

**Published:** 2020-08-25

**Authors:** Zhantao Deng, Shuai Wang, Mengyuan Li, Guangtao Fu, Chang Liu, Shuxian Li, Jiewen Jin, Feng-Juan Lyu, Yuanchen Ma, Qiujian Zheng

**Affiliations:** ^1^Department of Orthopedics, Guangdong Provincial People's Hospital, Guangdong Academy of Medical Sciences, Guangzhou 510080, China; ^2^South China University of Technology-The University of Western Australia Joint Center for Regenerative Medicine Research, School of Medicine, South China University of Technology, Guangzhou 510641, China; ^3^Department of Endocrinology, The First Affiliated Hospital, Sun Yat-sen University, Guangzhou 510080, China

## Abstract

**Aim:**

To investigate the different effects on osteolysis between commercial pure Ti particles and TiAl6V4 particles obtained from prosthesis of patients with aseptic loosening.

**Method:**

Scanning electron microscope, energy dispersive X-ray spectrometry, and X-ray diffraction were used for the size test, chemical composition test, and phase analysis of two kinds of Ti particles. Microcomputed tomography (micro-CT) and 3-dimensional reconstruction analysis were applied to analyze the bone loss quantitatively and radiologically. Hematoxylin-eosin (HE) staining and tartrate-resistant acid phosphatase (TRAP) staining were used to assess the histologic difference.

**Result:**

TiAl6V4 particles were constituted by FeO, Al_45_V_7_, and Al_3_Ti while pure Ti particles were constituted by Ti, Ti_3_O, and C_4_H_7_NO_3_. Similar particle size of nanoscale was detected of two Ti particles. A TiAl6V4 osteolysis model had more severe bone loss when scanned with micro-CT and assessed by quantitative analysis. TiAl6V4 also presented deeper and wider calvarial bone loss in HE staining and more activated osteoclasts in TRAP staining.

**Conclusion:**

A mouse calvarial model is the most effective animal model for the primary in vivo research of aseptic loosening. Compared with commercial Ti particles, TiAl6V4 particles derived from prosthesis of an aseptic loosening patient had more severe bone loss and more activated osteoclast, which was more consistent with pathogenesis of aseptic loosening *in vivo*, had high success rate of establishment of a model, and was more desired in animal modeling.

## 1. Introduction

Total joint replacement (TJR) is a highly successful procedure to manage the pain and disability resulted from osteoarthritis and fractures, and there are about 1.5 million TJR procedures performed worldwide each year [1]. Aseptic loosening is the most common long-term cause of TJR failure, but the underlying mechanism is still unclear [2]. The release of biomaterial wear particles from prosthesis, which caused inflammatory response in bone microenvironment, is a recognized pathogenesis [3]. Since it takes long time of follow-up to detect aseptic loosening in clinical cases, more studies focus on cellular and tissue mechanisms, in which valid *in vivo* models are urgently needed.

A series of animal models have been used for studying the biology of aseptic osteolysis, including sheep, dogs, and rabbits [4]. However, a mouse model is most widely used, owing to its low cost of maintenance and diverse options in immune and genetic features [5]. Three mouse models are now available, including the air pouch model, the calvarial model, and intramedullary implant models [5]. Due to the ability to test the host response in an orthotopic bone site, the rapidity in developing osteolysis, the availability of quantified images of bone loss, and the relatively low cost, the calvarial model is the most widely used for the study of particle-induced osteolysis [5].

Titanium (Ti), chrome-cobalt (Cr-Co), and polymethylmethacrylate (PMMA) particles are most commonly used in the studies of aseptic loosening [6]. Those particles are usually commercially available, which are different from the wear particles released from prosthesis in patients with aseptic loosening. Therefore, we compared the different effects on osteolysis between commercial pure Ti particles and TiAl6V4 particles obtained from the prosthesis of patients with aseptic loosening in the present study.

## 2. Methods

### 2.1. Preparation of Particles

**Figure 1:**
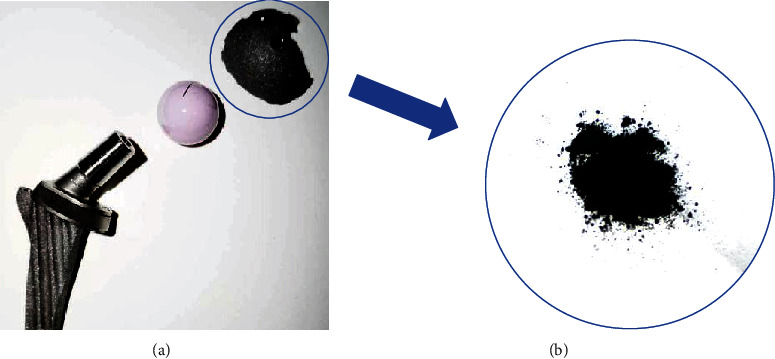
The prosthesis of patients with aseptic loosening (a) and TiAl6V4 particles (b) obtained from it.

TiAl6V4 particles were obtained from the prosthesis of patients with aseptic loosening. The TiAl6V4 prosthesis was placed in a fabricated high-vacuum three-electrode direct current under 10^−3^ Pa vacuum, 0.04 MPa argon and hydrogen 3 : 2 (*v*/*v*), and 650 A cathode current (Figure 1) [7]. The pure Ti particles were commercially available from the LINK Company. The particles were suspended in phosphate-buffered saline (PBS) at a concentration of 50 mg/mL as a stock solution.

### 2.2. Scanning Electron Microscope and Chemical Composition Test

The TiAl6V4 and pure Ti particles were first put into a vacuum drying oven (DZF-6012) at 133 Pa and 80°C for 12 h. After dehydration, the particle samples were adhered on the conductive adhesive, which were pasted on the tin plate. The samples were scanned by a field emission scanning electron microscope (Carl Zeiss AG, Merlin, Germany), and the images were obtained by assorted software. Chemical composition of tested particles was characterized by assorted energy dispersive X-ray spectrometry.

### 2.3. X-Ray Diffraction

The TiAl6V4 and pure Ti particles were sent to the Medical Device Research and Testing Center of South China University of Technology for phase analysis via X-ray diffraction (Malvern Panalytical, Empyrean, UK).

### 2.4. Murine Calvarial Osteolysis Model

The murine calvarial osteolysis model is widely used to study the pathogenesis of aseptic loosening. Six-week-old C57BL/J6 mice (6 mice for each group) were first anesthetized and placed in a prone position. Then, the skin of the cranium was incised along the line between the two eyes and ears, and the cranial periosteum was removed by sharp dissection. 50 *μ*L of metal particle (TiAl6V4 and pure Ti) suspension (50 mg/mL, in PBS) was embedded in the middle of the calvarias. 50 *μ*L of PBS was applied as a sham group. After 2 weeks, all the mice were sacrificed, and the calvarias were harvested for further experiments. The research protocol was approved by the ethics committee of Guangdong Provincial People's Hospital, China.

### 2.5. Microcomputed Tomography (micro-CT) and 3-Dimensional Reconstruction Analysis

The calvarial caps of PIO murine models were analyzed by micro-CT scanning (SkyScan, Belgium) as previously reported [8]. 550 mA and 45 kV of X-ray energy and 18 mm resolution were set in the scanning. Quantitative analysis was performed in region around the midline after 3-dimensional reconstruction by assorted software. The analysis parameters included bone mineral density (BMD), bone volume/total volume (BV/TV), total porosity percentage, trabecular number (Tb.N), trabecular thickness (Tb.Th), and trabecular separation/spacing (Tb.Sp).

### 2.6. Histologic Staining

The calvarias of PIO murine models were first decalcified in 15% EDTA-PBS solution and then embedded in paraffin and sliced in the midline area where particle deposits. Hematoxylin-eosin (HE) staining (Beyotime, C0105, China) was used to observe overall pathological characteristics. Tartrate-resistant acid phosphatase (TRAP) staining (Sigma, 387A, USA) was used to detect osteoclasts activation. All the histologic staining was photographed with a light microscope (Nikon, C2+, Tokyo, Japan).

### 2.7. Statistical Analysis

All the data was analyzed by the SPSS22.0 software (SPSS, Chicago, IL) and were presented as the mean ± standard error of the means. The differences between different groups were analyzed by the Brown-Forsythe test. *P* < 0.05 was considered as a significant difference.

## 3. Results

### 3.1. The Difference of Physical Characteristics between TiAl6V4 and Pure Ti Particles

**Figure 2:**
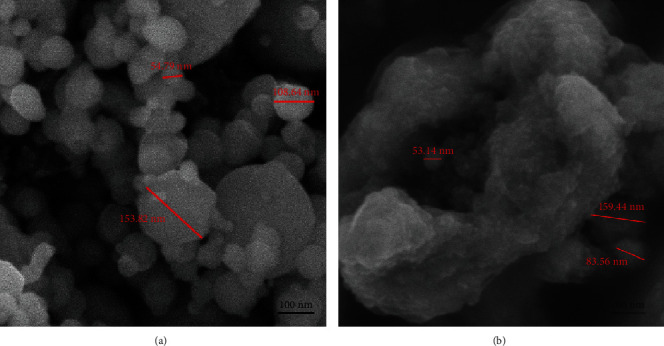
Scanning electron microscope of two Ti particles: (a) TiAl6V4 particles and (b) pure Ti particles.

**Figure 3:**
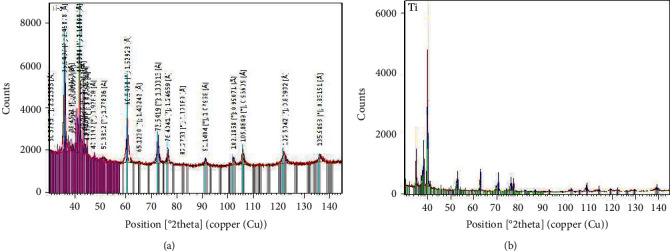
X-ray diffraction of two Ti particles: (a) TiAl6V4 particles and (b) pure Ti particles.

The size of metal particles appeared ranging between 54.79 nm and 153.82 nm for TiAl6V4 particles and 53.14 nm to 159.44 nm for pure Ti particles from scanning electron microscope images (Figure 2). The chemical compositions of TiAl6V4 and pure Ti particles are shown in Table 1. When analyzed with X-ray diffraction, the main phases of TiAl6V4 particles were constituted by FeO, Al_45_V_7_, and Al_3_Ti while pure Ti particles were constituted by Ti, Ti_3_O, and C_4_H_7_NO_3_ (Figure 3).

### 3.2. Radiologic Difference between Two Ti Particle-Induced Calvarial Osteolysis Models

**Figure 4:**
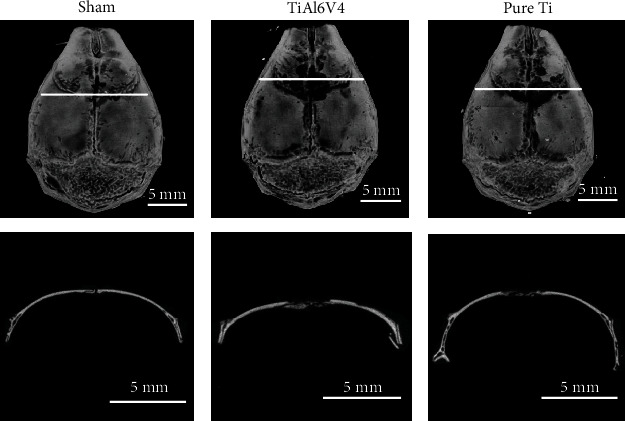
Microcomputed tomography with 3-dimensional reconstruction of calvarial caps from the murine osteolysis model induced by PBS (sham), TiAl6V4, and pure Ti particles. Three-dimensional reconstruction images appear in the first row, and the white horizontal line indicates the location of cross-sectional image in the second row.

**Figure 5:**
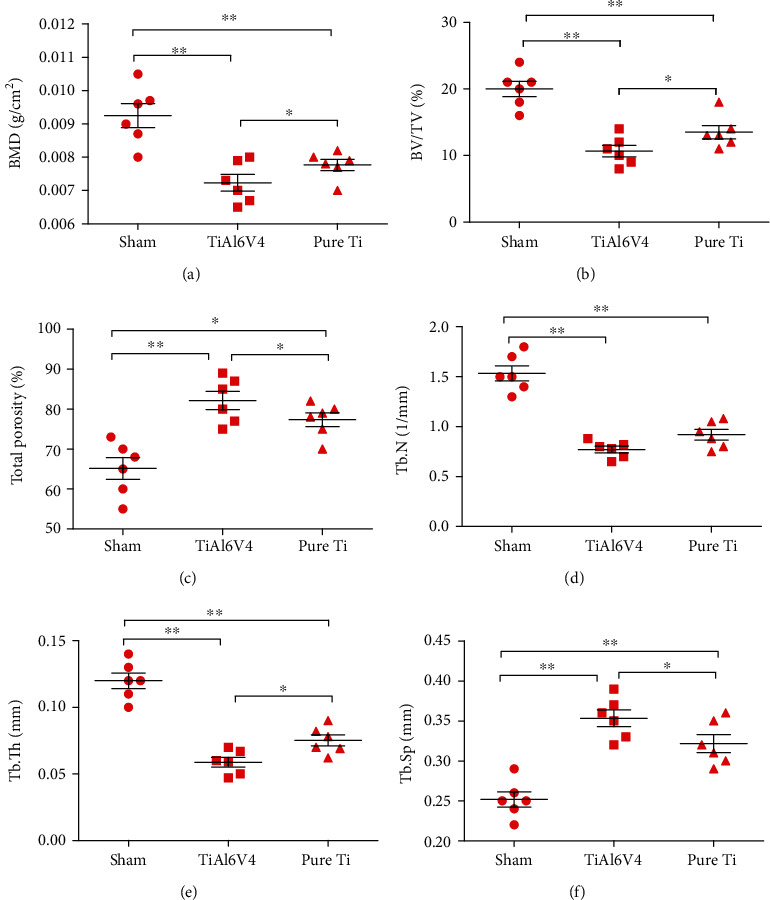
Quantitative analysis of osteolysis in calvarial caps from murine osteolysis model induced by PBS (sham), TiAl6V4, and pure Ti particles: (a) bone mineral density (BMD); (b) bone volume/total volume (BV/TV); (c) total porosity percentage; (d) trabecular number (Tb.N); (e) trabecular thickness (Tb.Th); (f) trabecular separation/spacing (Tb.Sp).

As shown from both 3-dimensional reconstruction images and representative coronal photographs at the cross-section of micro-CT scanning, significant bone loss was observed in both the TiAl6V4 and pure Ti groups when compared with the sham group (Figure 4). However, the TiAl6V4 group had more severe bone loss compared with the pure Ti group. Further quantitative analysis also revealed significant reduction of BMD, BV/TV, and Tb.Th and increase of total porosity and Tb.Sp in the TiAl6V4 group when compared with the pure Ti group (Figure 5).

### 3.3. Histologic Characteristics of Two Ti Particle-Induced Calvarial Osteolyses

**Figure 6:**
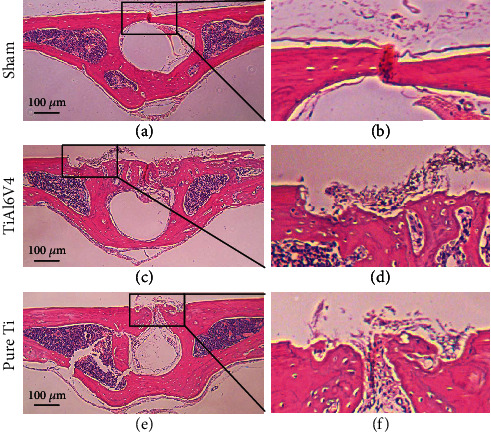
Representative images of HE staining of the calvaria from murine osteolysis model induced by PBS (sham), TiAl6V4, and pure Ti particles. The images in black box in (a, c, e) are enlarged and presented in (b, d, f).

**Figure 7:**
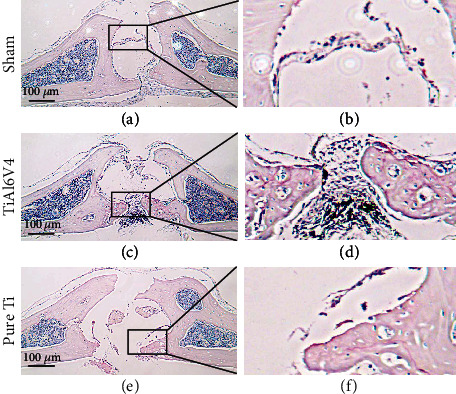
Representative images of TRAP staining of the calvaria from murine osteolysis model induced by PBS (sham), TiAl6V4, and pure Ti particles. The activated osteoclasts are stained as red. The images in black box in (a, c, e) are enlarged and presented in (b, d, f).

To further investigate the histologic difference of two osteolysis models, HE staining was applied. As shown in Figure 6, the TiAl6V4 particle-induced osteolysis model exhibited deeper and wider calvarial bone loss when compared with the pure Ti model. Furthermore, the osteoclasts were more activated in the TiAl6V4 group when staining with TRAP (Figure 7).

## 4. Discussion

The data from the present study revealed that compared with the commercial pure Ti osteolysis model, the TiAl6V4 osteolysis model had more severe bone loss when scanned with micro-CT and significant reduction of BMD, BV/TV, and Tb.Th and increase of total porosity and Tb.Sp after further quantitative analysis. Regarding histologic characteristics, TiAl6V4 also presented deeper and wider calvarial bone loss in HE staining and more activated osteoclasts in TRAP staining.

Wear particle-induced aseptic loosening is considered the main cause of long-term failure in TJR, which leads to surgical revision and huge economic burden in public health. In this regard, thorough understanding of pathophysiology of aseptic loosening is vital for therapeutic development, and appropriate animal models are essential for the researches. A series of animals have been used for this purpose, including dogs, sheep, rabbits, and mice. In a large animal model (including dogs and sheep), total hip arthroplasty is usually applied, and different kinds of alloy femoral component are implanted. The animals were allowed full ambulation postoperatively and sacrificed after 26-52 weeks [9, 10]. For the rabbit model, both total hip arthroplasty and drug test chamber, which are implanted at the level of the cortex in the proximal medial tibial metaphysis and connected with a diffusion pump, were used [11]. By simulating the TJR procedure on animal models, the behavior of the implant in the host is able to be understood globally. However, since the high cost of maintenance, long term in modelling, and difficulties in therapeutic interventions, they are not the most used animal models for primary *in vivo* research [5]. A mouse model, owing to its low cost of maintenance, short term in modelling, and the facility to reach sufficient numbers of subjects to strengthen statistical results, is the most effective alternative [12].

Two main mouse methods are mostly used for wear particle-induced osteolysis, namely, air pouch and calvarial models. In the air pouch model, subcutaneous space, which is called as the air pouch, is first established. Then, wear particles (including metal and polymeric biomaterial) are introduced in the air pouch, as well as implantation of the bone tissue [13]. This model is mainly served as an initial proof of concept in the targeting of anti-inflammatory or antiresorptive events related to particle-induced osteolysis [5]. However, since the bone implantation is nonvascular and has no biological activity, it is difficult to assess the direct interactions between particles and the bone tissue. The calvarial osteolysis model has several superiorities over the air pouch model. First, the calvarium is vascular, and the particles are exposed to the calvaria directly, which makes it possible to assess interactions between wear particles and bone homeostasis [14, 15]. Second, micro-CT or histologic staining can be used to quantitatively assess the bone loss, both radiologically and biologically [16]. Third, transgenic and gene knockout mice can be used to investigate the relationship between wear particles and bone loss [17, 18].

Previous studies often focus on the methods of modeling; the sources of wear particles are seldom investigated. In the present study, we explored the difference of different sources of particles, including commercially purchased and derived from prosthesis of aseptic loosening. Our data shown that the TiAl6V4 model had deeper and wider calvarial bone loss and more activated osteoclast when compared with 0the commercial pure Ti model, in spite of similar particle size of nanoscale. The TiAl6V4 particles derived from Ti prosthesis of aseptic loosening patient had more complicated components when compared with commercial one, which may account for the different biological response of calvarial osteolysis model in part. Aluminum had toxicity on bone metabolism, including collagen synthesis and matrix mineralization [19]. The application of aluminum also reduced elastic modulus and stress, indicators of bone material intrinsic properties [20]. The exposure of metal ions (including titanium, aluminum, and vanadium) led to a reduction in cell viability, higher rates of early apoptosis, and upregulated expression of inflammatory cytokines and RANKL in osteoblasts [21]. Our previous study used TiAl6V4 and Cr-Co particles derived from the prosthesis of aseptic loosening patient for the osteolysis model and found novel mechanisms and potential treatment of aseptic loosening, such as Sirtuin 1-mediated protein acetylation [8, 22], autophagy [16], and hydrogen sulfide [23]. The biological responses of different sources of wear particles need further investigation.

In conclusion, aseptic loosening is the most common long-term cause of TJR failure, in which cellular and tissue mechanisms are not clear and valid *in vivo* models are urgently needed. A mouse calvarial model is the most effective animal model for the primary in vivo research of aseptic loosening. Compared with commercial Ti particles, TiAl6V4 particles derived from prosthesis of an aseptic loosening patient had more severe bone loss and more activated osteoclast, which was more consistent with pathogenesis of aseptic loosening in vivo, had high success rate of establishment of model, and was more desired in animal modeling.

## Figures and Tables

**Table 1:** Chemical composition of TiAl6V4 and pure Ti particles.

Element	TiAl6V4		Pure Ti	
	Normalized mass (%)	Atomic percent (%)	Normalized mass (%)	Atomic percent (%)
Ti	28.70	9.78	67.52	37.64
C	57.84	78.57	10.39	23.11
N	–	–	10.97	20.90
O	10.22	10.42	10.83	18.07
Si	0.03	0.02	0.29	0.27
Al	0.73	0.44	—	—
V	1.92	0.62	—	—
Co	0.55	0.15	—	—

## Data Availability

All data, models, and code generated or used during the study appear in the submitted article.
